# Long non-coding RNA AK006774 inhibits cardiac ischemia-reperfusion injury via sponging miR-448

**DOI:** 10.1080/21655979.2021.1954135

**Published:** 2021-08-08

**Authors:** Shen Nie, Xiaoya Cui, Jinping Guo, Xiaohua Ma, Haijun Zhi, Shilei Li, Yong Li

**Affiliations:** Department of Emergency, Cangzhou Central Hospital, Cangzhou, China

**Keywords:** lncRNA AK006774, ischemia-reperfusion injury, miR-448

## Abstract

In recent years, the incidence and mortality of myocardial infarction (MI) have been increasing throughout the world, threatening public health. Non-coding RNAs (ncRNAs), including microRNAs (miRNAs) and long non-coding RNAs (lncRNAs), play critical roles in the progression of MI. The present study aimed to investigate the role of lncRNA AK006774 in the progression of myocardial infarction and find out novel therapeutic or diagnostic target of myocardial infarction. A mouse ischemia/reperfusion (I/R) model and 2,3,5-Triphenyte-trazoliumchloride (TTC) staining were performed to evaluate the effects of AK006774 on I/R injury *in vivo*. Hypoxia/reoxygenation (H/R) models using primary cardiomyocytes have been established. Flow cytometry and Terminal Deoxynucleotide Transferase dUTP Nick End Labeling (TUNEL) assays were performed to evaluate the effects of AK006774 on cardiomyocyte apoptosis. Luciferase and RNA pull-down assays were performed to verify the interaction between miR-448 and its targets. Western blotting and quantitative PCR were performed to determine protein and gene expression, respectively. We first found that AK006774 overexpression reduced I/R-induced infarct area and cardiomyocyte apoptosis *in vivo*. Accordingly, AK006774 inhibited apoptosis and oxidative stress in cardiomyocytes subjected to H/R treatment *in vitro*. Mechanistically, AK006774 modulated the expression of bcl-2 by sponging miR-448. Overexpression of miR-448 antagonized the effects of AK006774 on cardiomyocyte apoptosis. The AK006774/miR-448/bcl-2 signaling axis acts as a key regulator of I/R injury and may be a potential therapeutic or diagnostic target for the treatment of MI.

## Graphical abstract

**Figure:**
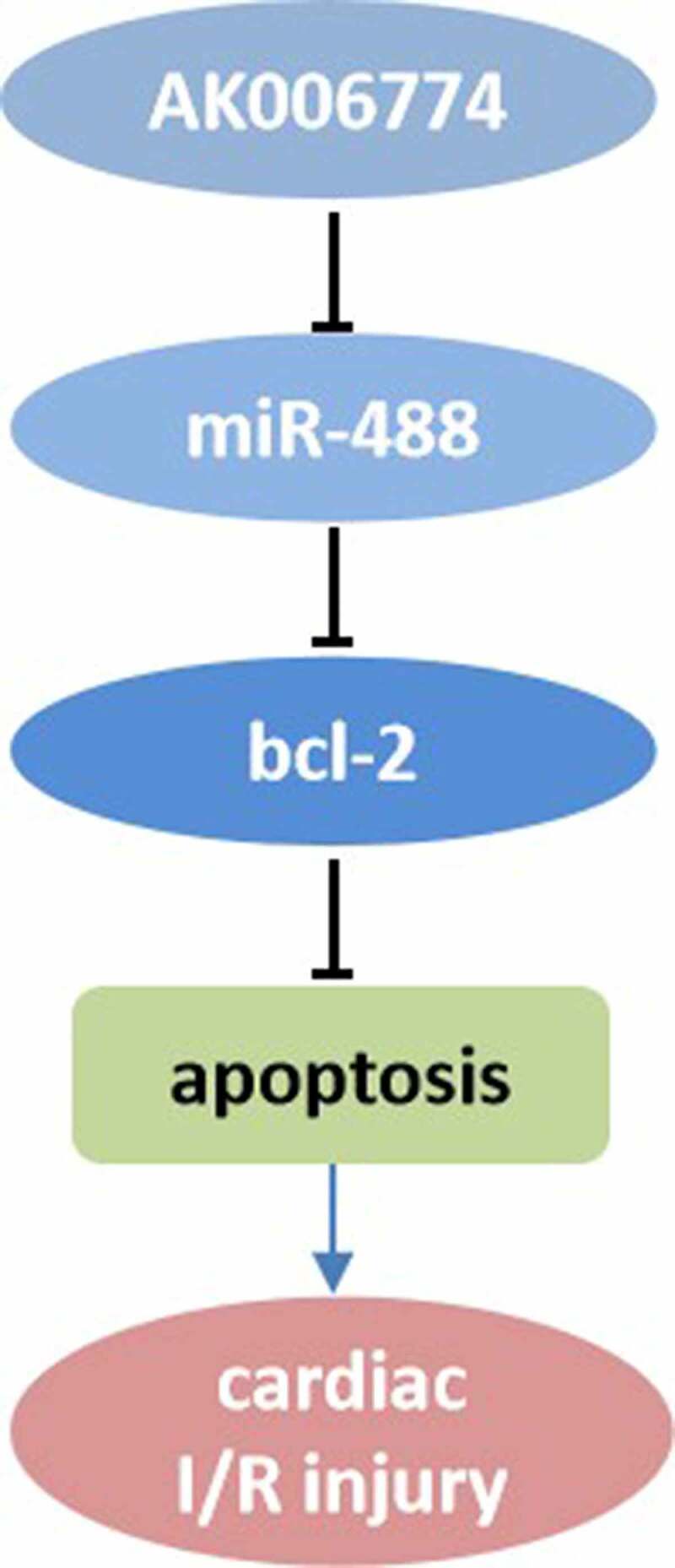

## Introduction

Myocardial infarction (MI) is the leading cause of heart failure worldwide [1,2]. Myocardial ischemia/reperfusion (I/R) injury refers to metabolic dysfunction caused by reperfusion of ischemic myocardial blood flow and aggravation of structural damage of cardiomyocytes, resulting in cell death and enlargement of infarction [3]. Various factors complicate the progression of MI such as coronary artery bypass grafting reperfusion therapy, coronary artery thrombolysis, and percutaneous coronary intervention that increase the incidence and mortality of cardio-cerebrovascular diseases [4]. Therefore, elucidating the molecular mechanism of cardiac I/R injury is of great importance to find out specific molecular target that has good therapeutic effect and few side effects.

Long non-coding RNAs (lncRNAs) are a class of non-coding RNAs with a length of more than 200 nucleotides [5]. lncRNAs regulate gene expression at multiple levels including epigenetic regulation, transcription, and post-transcriptional regulation. Many studies have shown that lncRNAs are closely related to myocardial I/R injury. lncRNAs may be potential biomarkers for the pathological mechanism of myocardial ischemia-reperfusion injury and may provide effective therapeutic targets [6–8].

Many previous studies have revealed the critical function of lncRNAs in the progression of cardiovascular diseases such as cardiac I/R injury, via binding to microRNAs (miRNAs) [7–9]. For instance, lncRNA TUG1 knockdown inhibits I/R-induced myocardial injury by altering miR-142-3p expression and autophagy [9]. lncRNA H19 ameliorates cardiac I/R injury by targeting miR-22-3p [10]. lncRNA CAIF blocks p53-mediated myocardin transcription and inhibits autophagy-relevant myocardial infarction injury [11]. However, the precise mechanisms underlying the effects of lncRNAs on MI remain to be elucidated.

MiRNAs are small, non-coding RNAs that mediate gene expression by influencing the stability and translation of mRNAs. It has been shown that miRs regulate biological functions such as proliferation, migration, invasion and apoptosis of cells. Previous studies indicated that miR-143-3p mediates the protective effect of LINC00528 silencing on cardiac infarction [12]. MicroRNA-21 prevents excessive inflammation and cardiac dysfunction after myocardial infarction through targeting KBTBD7 [13].

In this study, we found that AK006774 was upregulated after cardiac I/R. We hypothesis that AK006774 must play critical role in the development of cardiac I/R injury. The aims and goals were to explore the effect and mechanism of AK006774 in cardiac I/R injury. We firstly found that AK006774 could reduce the size of the infarct area and cardiomyocyte apoptosis *in vivo* and decreased the apoptosis of cardiomyocytes after H/R *in vitro*. In addition, AK006774 functioned as a ceRNA to regulate the expression of bcl-2 vis bound with miR-448, which further targets bcl-2 and modulates its expression. These findings extend our understanding of the function of lncRNAs and provide novel therapeutic targets for the treatment of cardiac I/R injury.

## Materials and methods

### Plasmids construction

Adenoviruses overexpressing AK006774 (Ad-AK006774) and control vector (Ad-nc) were synthesized by Hanbio (Shanghai, China). The miR-448 mimic and inhibitor, along with their negative controls, were synthesized by General Bio Company (Hefei, Anhui). Wild-type and mutant sequences containing the binding sites of miR-448 targeting AK006774 and bcl-2 were synthesized and cloned into the pGL3 reporter plasmid (Promega, USA). These oligonucleotides were transfected into cells using Lipofectin2000 (Thermo Fisher Scientific), USA kit according to the instructions [14].

### I/R model establishment

The 12-week-old female C57BL/6 mice were purchased from Huafukang (Beijing, China). The mice were housed on a 12-h light/dark cycle at 22–26°C and 50–70% humidity, with free access to water and food. All *in vivo* studies were approved by the Animal Studies Committee of Cangzhou Central Hospital and were performed following institutional guidelines.

Mice were intravenously injected with Ad-AK006774 or Ad-nc at a dose of 1 × 10^9^ PFU for three consecutive days. I/R injury was then inflicted on C57BL/6 mice. Briefly, the fourth intercostal space was opened to expose the heart. Thereafter, ischemia was induced for 40 min by ligating the left coronary artery with a 7–0 suture. Then, the coronary artery was released by loosening the suture for 24 h. Finally, the mice were sacrificed to obtain the heart tissue for further studies [15].

### 2,3,5-Triphenyte-trazoliumchloride (TTC) staining

For TTC staining, 2% Evans blue was injected into the heart from the femoral vein after ligation of the left anterior descending (LAD) coronary artery. Heart tissues were collected immediately after injection and rinsed with ice-cold normal saline. The heart was frozen at −20°C for 30 min and then transversely cut into 1 mm-thick slices. The tissues were then incubated with TTC (Solarbio, Beijing, China) at 37°C for 15 min. After fixation with formaldehyde for 24 h, the heart slices were visualized. The white area was considered the infarct area and the red area was considered the area at risk (AAR) [15].

### Primary culture of cardiomyocytes

Neonatal mouse cardiomyocytes (NRMCs) were separated from 1-day-old C57BL/6 mice. The ventricles of hearts were cut and minced into 1 mm^3^ pieces and digested with 0.2% collagenase II for 2 h on ice. Digested tissues were pipetted and strained with a 70 μm strainer. Cells were collected and cultured in DMEM supplemented with 10% fetal bovine serum [14].

### Hypoxia/reoxygenation (H/R)

Cardiomyocytes were cultured in a hypoxic medium with a mixed gas consisting of 5% CO_2_ and 95% N_2_ at 37°C for 4 h and then cultured in a fresh culture medium with a mixed gas consisting of 95% air and 5% CO_2_ for 12 h. The cells of the control group were cultured under normal conditions with 95% O_2_/5% CO_2_ at 37°C [16].

### Terminal Deoxynucleotide Transferase dUTP Nick End Labeling (TUNEL) assay

The TUNEL assay was performed using a detection kit (Roche Applied Science, Mannheim, Germany). The heart tissues were rinsed, dehydrated, embedded in paraffin, and cut into 4 μm thick slices. The experimental procedures were performed following the manufacturer’s instructions. Slices were mounted, and the number of TUNEL-positive nuclei was calculated. The percentage of apoptotic cells to total cardiomyocytes was then calculated [16].

### Flow cytometry

Approximately 5 × 10^6^ cells/mL were collected, centrifuged at 800 × g for 5 min, and the culture medium was discarded. Then, the cells were washed once with 3 mL phosphate buffered saline (PBS). After centrifugation in PBS, the cells were fixed with 70% ethanol at 4°C for 2 h. The fixative was discarded by centrifugation, and cells were resuspended in 3 mL PBS for 5 min. Thereafter, cells were stained with 1 mL AnnexinV-FITC and propidium iodide (PI, Jiancheng, Nanjing, China) in the shade at 4°C for 30 min. Cell apoptosis was detected by flow cytometry using a BD FACSVerse system (BD Science, USA) [16].

### Malondialdehyde (MDA) and lactate dehydrogenase (LDH) evaluation

The cardiomyocytes were collected and centrifuged at 3,000 × g for 10 min at 4°C. The supernatant was collected and the amounts of LDH and MDA were detected using commercial kits following the manufacturer’s instructions (Beyotime, Shanghai, China) [17].

### Reactive oxygen species (ROS) evaluation

After treatment, the cardiomyocytes were incubated with 2,7-dichlorofluorescein diacetate (Jiancheng, Nanjing, China) at 37°C for 40 min. The mean fluorescence intensity was detected using an automatic fluorescence microplate reader (Bio-Rad, USA) [17].

### Quantitative real-time PCR (qPCR)

RNA was extracted using TRIzol reagent (Invitrogen, Carlsbad, CA, USA) following the manufacturer’s protocols. RNA (1 μg) was reverse transcribed into cDNA using a reverse transcription kit (Transgen, Beijing, China). Thereafter, 10 ng of cDNA was used as the template for qPCR experiments. The relative expression of genes was evaluated using SYBR green mix (Yisheng, Shanghai) in a 7500 Fast Real-time PCR system (Applied Biosystems, USA). The reaction was incubated in 96-well plates at 95°C for 30 sec, followed by 45 cycles of amplification at 95°C for 5 sec and 63°C for 30 sec. The relative expression was calculated using the 2-∆∆CT method. U6 was the internal control for miR-448, while GAPDH was the house keeping gene for AK006774 and other mRNAs. The primers were provided by Genewiz (Beijing, China) [17].

### Pull-down assay with biotinylated miRNA

Biotin-labeled miR-448 probes or negative controls were obtained from Thermo Fisher Scientific (CA, USA). Cardiomyocytes were transfected with probes. After 48 h, the cardiomyocytes were harvested and lysed. The lysates were then incubated with pre-coated beads overnight. RNA enrichment was evaluated using a qPCR assay [18].

### Luciferase assay

Cardiomyocytes were inoculated into 96-well plates and cultured to 60% cell density before transfection. The wild-type (WT) or mutant (MUT) AK006774 or bcl-2 fragments were inserted into the Xba1 restriction site of the pGL3 luciferase vector (Genechem, Shanghai, China). Cells were co-transfected with 600 ng pGL3 vector and 20 nmol miR-448 mimic or mimic control. After incubation for 48 h, firefly and renilla luciferase activities were detected using a dual-luciferase system (Promega, USA). Firefly luciferase activity was measured for internal control using 100 μL luciferase assay reagent II (Luciferase Assay Reagent, Promega, USA). Finally, differences between firefly and renilla luciferase activities were calculated to determine the relative luciferase activity [18].

### Statistical analysis

All data are presented as the mean ± standard deviation (SD). Statistical analysis was performed using SPSS 20 software (Abbott Laboratories, Chicago, IL, USA). Non-paired Student’s t-test was applied to determine the difference between two groups, and one-way ANOVA for multiple groups. Pearson’s correlation analysis was also performed. P-value < 0.05 was considered statistically significant.

## Results

Here, we found that AK006774 was upregulated after cardiac I/R. We hypothesis that AK006774 must play critical role in the development of cardiac I/R injury. The aims and goals were to explore the effect and mechanism of AK006774 in cardiac I/R injury. In vivo and in vitro I/R model using mouse and cardiomyocytes respectively were established to evaluate the effect of AK006774 on I/R injury. Luciferase and RNA pull down assays were used to detect the targeting between miR-448 and its targets. Rescue experiments were carried out to further confirm the interaction between AK006774 and miR-448.

### *AK006774 is downregulated in MI* in vivo *and* in vitro

**Figure 1.**
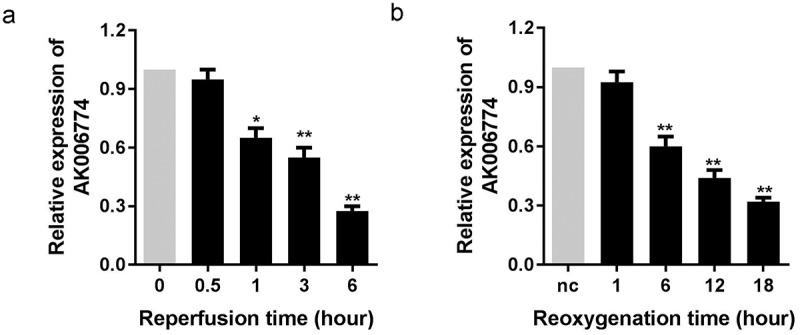
AK006774 is downregulated in the myocardium and cardiomyocyte subjected to I/R or H/R. (a) The expression of AK006774 in the myocardium after different times of reperfusion was evaluated by qPCR. (b) The expression of AK006774 in cardiomyocytes after different times of oxygenation was detected by qPCR. *p < 0.05 vs 0 h or nc group, **p < 0.01 vs 0 h or nc group

We first evaluated the expression level of AK006774 in the myocardium and cardiomyocytes after different reperfusion or reoxygenation periods using qPCR. As shown in Figure 1a and b, AK006774 was downregulated significantly 1 h after reperfusion in the myocardium and 6 h after oxygenation in cardiomyocytes (Figure 1a and b).

### AK006774 inhibits H/R induced apoptosis and oxidative stress of cardiomyocytes

**Figure 2.**
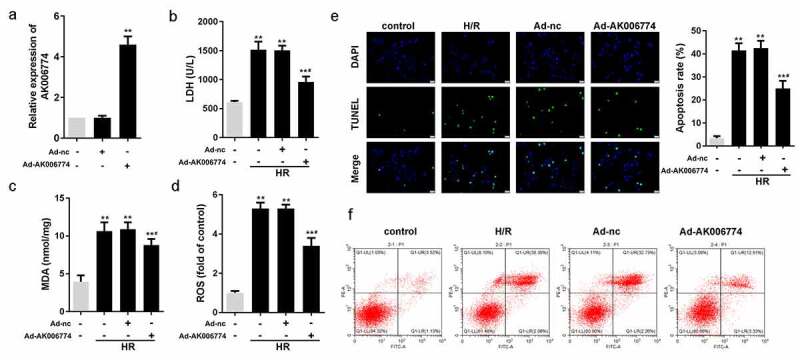
AK006774 inhibits the H/R-induced oxidative stress and apoptosis *in vitro*. (a) qPCR was used to evaluate the efficiency of AK006774-overexpressing adenovirus. (b-d) The level of LDH, MDA, and ROS were detected using commercial kits. (e) TUNEL staining was carried out to detect the apoptosis of cardiomyocytes. (f) Flow cytometry and Annexin V/PI cardiomyocytes staining were performed to detect the apoptosis of cardiomyocytes. *p < 0.05 vs control group, **p < 0.01 vs Ad-nc or control group, ^#^p < 0.05 vs H/R+ Ad-nc group

An *in vitro* H/R model was established to investigate the function of AK006774. First, the efficiency of the adenovirus overexpressing AK006774 was detected by qPCR, indicating a good over-expression effect of Ad-AK006774 (Figure 2a). We detected changes in MDA, LDH, and ROS levels in cardiomyocytes. H/R notably increased the amount of MDA, LDH, and ROS, while AK006774 remarkably reversed this elevation (Figure 2b–d). AK006774 notably decreased the apoptosis of cardiomyocytes induced by H/R treatment (Figure 2e). Moreover, flow cytometry revealed a similar finding with the TUNEL assay, indicating that AK006774 overexpression inhibited H/R-induced apoptosis (Figure 2f).

### *AK006774 inhibits cardiac I/R injury* in vivo

**Figure 3.**
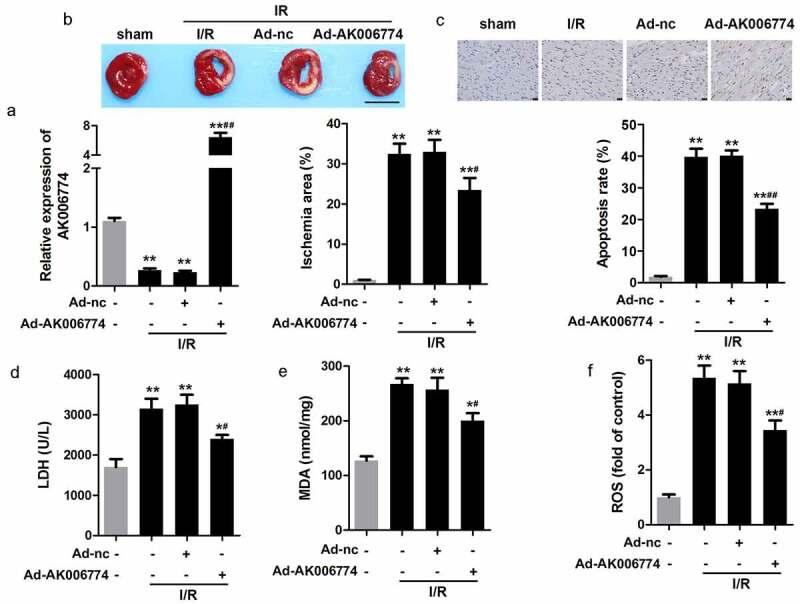
AK006774 inhibits the I/R-induced oxidative stress and cardiac injury *in vivo*. (a) qPCR was used to evaluate the efficiency of AK006774-overexpressing adenovirus. (b) TTC staining was performed to evaluate the infarct area of the heart. (c) TUNEL staining was carried out to detect the apoptosis of cardiomyocytes. (d–f) The levels of LDH, MDA, and ROS in the myocardium were detected using commercial kits. ** p < 0.01 vs Ad-nc or sham group, ^#^ p < 0.05 vs I/R+ Ad-nc

To further verify the function of AK006774 in I/R injury, we used an I/R model in mice. Again, we found that adenovirus overexpressing AK006774 significantly increased AK006774 levels in the myocardium (Figure 3a). TTC staining showed that AK006774 decreased the infarct area compared to the I/R group (Figure 3b). TUNEL assay was performed to evaluate apoptosis in the myocardium. The results revealed that AK006774 treatment decreased myocardial apoptosis induced by I/R (Figure 3c). Moreover, MDA, LDH, and ROS were detected in the myocardium. *In vivo* results paralleled the *in vitro* studies, showing that AK006774 reduced the elevation of MDA, LDH, and ROS in comparison with the I/R group (Figure 3d–f).

### miR-448 is a target of AK006774 in cardiomyocytes

**Figure 4.**
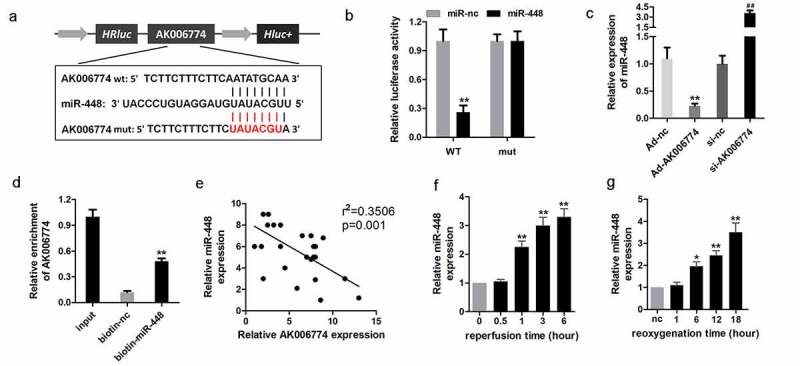
miR-448 is a target of AK006774 in cardiomyocytes. (a) The potential binding sequence between AK006774 and miR-448 was predicted by bioinformatics analysis. (b) A luciferase reporter assay was performed to confirm the interaction between miR-448 and AK006774. (c) qPCR was used to evaluate the level of miR-448 in different groups. (d) RNA pull-down was used to confirm the interaction between AK006774 and miR-448. (e) Pearson correlation analysis was performed to detect the correlation between AK006774 and miR-448. (f) The expression of miR-448 in myocardium after 30 min of ischemia and then different times of reperfusion was evaluated by qPCR. (g) The expression of miR-448 in cardiomyocytes after 4 h of hypoxia and subsequently different times of reoxygenation was detected with qPCR. * p < 0.05 vs miR-nc or biotin-nc, ** p < 0.01 vs miR-nc, ^##^ p < 0.01 vs si-nc group

It is well known that lncRNAs are capable of sponging miRNAs. Thus, we further explored the target miRNAs of AK006774. MIRDB databases were used to predict the miR targets of AK006774, and miR-448 was selected as a potential target. Figure 4a shows the target region between AK006774 and miR-448. A luciferase assay was performed to verify the binding between AK006774 and miR-448. The results revealed that miR-448 notably decreased the luciferase activity when co-transfected with pGL3-wt-AK006774 but not the pGL3-mut-AK006774 vector (Figure 4b). The qPCR analysis further indicated that AK006774 overexpression reduced the level of miR-448 while silencing AK006774 elevated miR-448 expression (Figure 4c). RNA pull-down assay was conducted to further determine whether AK006774 could endogenously bind with miR-448. The results suggested that the biotin-labeled miR-448 probe enriched AK006774, indicating binding between AK006774 and miR-448 (Figure 4d). Pearson correlation analysis was used to detect the correlation between AK006774 and miR-448. As expected, AK006774 and miR-448 were negatively correlated (Figure 4e).

### MiR-448 is upregulated in cardiac I/R injury

To further elucidate the role of miR-448, we evaluated the level of miR-448 in I/R injury *in vivo* and *in vitro*. The results indicated that miR-448 was significantly upregulated in the myocardium 1 h after I/R and in cardiomyocytes 6 h after reoxygenation (Figure 4f and g).

### MiR-448 reverses the effect of AK006774 on cardiomyocyte apoptosis

**Figure 5.**
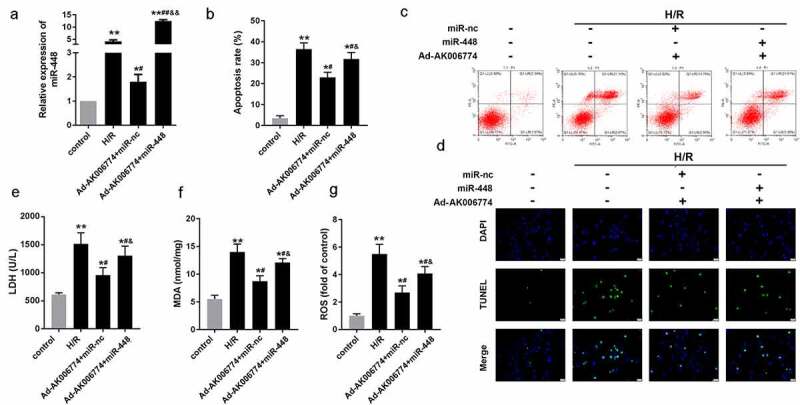
MiR-448 reversed the effect of AK006774 on cardiac I/R injury *in vitro*. (a) qPCR was performed to assess the level of AK006774 in cardiomyocytes in different groups. (b, c) Flow cytometry and Annexin V/PI staining were performed to detect the apoptosis of cardiomyocytes. (d) TUNEL staining was carried out to detect the apoptosis of cardiomyocytes. (e–g) The level of LDH, MDA, and ROS were detected using commercial kits. *p < 0.05 vs control,**p < 0.01 vs control, ^#^ p < 0.05 vs H/R group, ^##^ p < 0.01 vs H/R group, ^&^ p < 0.05 vs AK006774+ miR-nc group, ^&&^p < 0.01 vs AK006774+ miR-nc group

We performed rescue experiments to further confirm that AK006774 functions as a sponge of miR-448 in cardiomyocytes. Cardiomyocytes were transfected with adenovirus overexpressing AK006774 and miR-448 mimic along with their negative controls. AK006774-induced downregulation of miR-448 was alleviated by overexpression of miR-448 (Figure 5a). TUNEL assay and flow cytometry were performed to evaluate cell apoptosis. We observed that AK006774 significantly reduced the apoptosis rate induced by H/R, while miR-448 remarkably reversed this effect of AK006774 (Figure 5b–d); again, MDA, LDH, and ROS were detected. As expected, miR-448 reversed the effect of AK006774 on elevating the release of MDA, LDH, and ROS (Figure 5e–g).

### MiR-448 directly targets bcl-2 in cardiomyocytes

**Figure 6.**
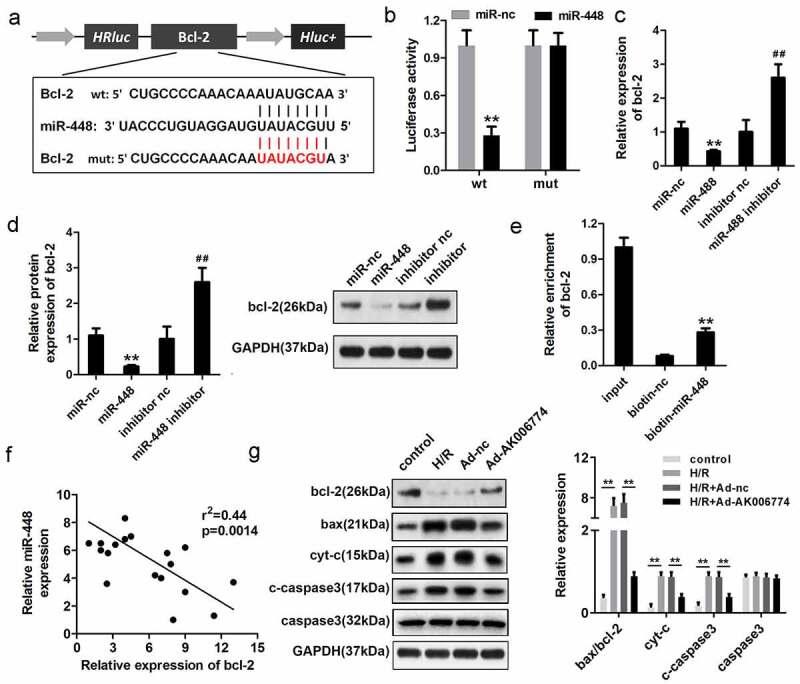
MiR-448 directly targets bcl-2 in cardiomyocytes. (a) The potential targeting region between miR-448 and bcl-2 was predicted by bioinformatics analysis. (b) A luciferase reporter assay was conducted to confirm the binding sequence between miR-448 and bcl-2 in cardiomyocytes. (c) qPCR was used to detect the mRNA level of bcl-2. (d) Western blot was used to detect the expression of bcl-2. (e) RNA pull-down assay was used to detect the interaction between miR-448 and bcl-2. (f) Pearson analysis was performed to investigate the correlation between miR-448 and bcl-2. (g) Western blot was used to evaluate the expression of bcl-2, cyt-c, and cleaved caspase3. **p < 0.01 vs miR-nc or biotin-nc,^#^p < 0.05 vs inhibitor nc group, ^##^p < 0.01 vs inhibitor nc group

Figure 6a shows the target region between miR-448 and bcl-2. Luciferase assays were carried out in cardiomyocytes. The results demonstrated that the miR-448 mimic significantly reduced the luciferase activity when co-transfected with pGL3-3ʹUTR-wt-bcl-2 but not with pGL3-3ʹUTR-mut-bcl-2 (Figure 6b). The qPCR analysis further indicated that miR-448 decreased the mRNA level of bcl-2, while knockdown of miR-448 promoted that of bcl-2 (Figure 6c). Accordingly, miR-448 reduced the protein expression of bcl-2, whereas silencing of miR-448 promoted the expression of bcl-2 (Figure 6d). RNA pull-down was conducted to investigate whether miR-448 could bind to bcl-2. The biotin-labeled probe of miR-448 enriched bcl-2, indicating a positive binding relationship between miR-448 and bcl-2 (Figure 6e). We performed Pearson’s analysis to detect the correlation between miR-448 and bcl-2; the results indicated a negative correlation between miR-448 and bcl-2 (figure 6f). To confirm the ceRNA relationship between AK006774 and bcl-2, we evaluated the expression of bcl-2 under H/R and AK006774 overexpressing treatment and found that AK006774 promoted the expression of bcl-2 but inhibited that of cyt-c and cleaved caspase3 (Figure 6g).

## Discussion

The present study identified that lncRNA AK006774 was downregulated in I/R treated myocardium and cardiomyocytes subjected to H/R. AK006774 overexpression remarkably reduced the size of the infarction area and myocardial apoptosis. An *in vitro* data indicated that AK006774 protected cardiomyocytes from H/R-induced cell apoptosis and oxidative stress

Evidence has shown that lncRNAs sponge miRNAs and block their inhibitory effect on the expression of downstream genes. Accordingly, GAS5, HOTAIR, TUG1, and NEAT1 have been reported to mediate cardiac I/R injury by interacting with miR-137, miR-126, miR-142-3p, and miR-27b [19–22]. In our work, we attempted to determine the miR sponged by AK006774 to elucidate its mechanism. Starbase software was used to predict the miR sponged by AK006774. Luciferase and RNA pull-down assays verified that AK006774 could sponge miR-448 and attenuate its expression. MiR-448 has been proved to be sponged by lncRNAs. For instance, LncRNA SNHG1 regulates the differentiation of Treg cells and affects the immune escape by sponging miR-448 in breast cancer [23]. Knockdown of Linc00052 alleviated spinal nerve ligation-triggered neuropathic pain via targeting miR-488 [24]. In addition, XIST, PVT1, NEAT1 and LncRNA PITPNA-AS1 were also demonstrated to be capable of sponging miR-488.

Most studies on miR-448 have focused on cancer research. For instance, miR-448 functions as a tumor suppressor in non-small cell lung cancer [25], pancreatic cancer [26], glioma [27], and osteosarcoma. In addition, previous studies indicated that miR-448 knockdown can attenuate spinal cord ischemia/reperfusion injury by modulating SIRT expression [28]. In the cardiovascular system, a comprehensive bioinformatics analysis of genes and miRNAs was performed to elucidate which factors may be involved in the progression of cardiac hypertrophy, demonstrating that miR-448 has a regulatory effect on differentially expressed SIM2 in TAC-induced hypertrophy [29]. These findings demonstrate the critical role of miR-448 in cardiovascular diseases. In the present study, we found that miR-448 reversed the protective effect of AK006774 on cardiac I/R injury, thus demonstrating the vital role of miR-448 in I/R injury. However, this alone is not sufficient to clarify the mechanism underlying the role of AK006774 or miR-448. Therefore, we focused on the downstream gene of miR-448.

MiR-448 has been reported to target numerous genes such as six1 [30], ZEB1 [31], and IGF1R [32]. We carried out bioinformatics prediction along with a luciferase assay and verified bcl-2 as a target gene of miR-448. It is well known that cell apoptosis and subsequent cell loss are the main causes of heart disease, inducing heart injury. Interestingly, bcl-2 is a key protein that regulates the initiation or progression of cell apoptosis [33]. The bcl-2 family plays a key role in mediating intrinsic or mitochondrial apoptosis [34–36]. The bcl-2 family of proteins including pro-apoptotic initiators, pro-apoptotic effectors, and anti-apoptotic proteins interact with one another to balance the apoptosis of cells [37–39]. The mechanism underlying the anti-apoptotic activity of the bcl-2 family proteins is mainly dependent on their binding to the pro-apoptotic BH-3-only proteins, thus preventing mitochondrial membrane permeabilization and the release of cyt-c into the cytoplasm. The limitation of this study is that we did not assess mitochondrial membrane permeabilization.

The first clinical therapeutic drug ‘Onpattro’ has been approved by FDA at 2018 based on RNA interference theory. If we find a specific and efficient target. It may significantly improve the treatment of the cardiac infarction disease. However, the precise mechanism underlying the progression of I/R injury remain to be elucidated. Much more work should be carried out to improve our understanding.

## Conclusion

We first indicated that AK006774 is downregulated in MI *in vivo* and *in vitro*. Overexpression of AK006774 inhibited the progression of MI by regulating the miR-448/bcl-2 axis. The AK006774/miR-448/bcl-2 signaling axis acts as a key regulator of I/R injury and may be a potential therapeutic target for the treatment of MI.

## Highlights

1. Silencing of lncRNA AK006774 which is upregulated in cardiac ischemia reperfusion can alleviate the I/R injury
2. AK006774 sponges miR-488 in cardiomyocytes
3. miR-448 targets bcl-2 in cardiomyocytes
